# Progress in the treatment of osteoarthritis with umbilical cord stem cells

**DOI:** 10.1007/s13577-020-00377-z

**Published:** 2020-05-23

**Authors:** Hanguang Liang, Haiqiang Suo, Zhiwei Wang, Wei Feng

**Affiliations:** Department of Bone and Joint, The First Hospital of Jilin University, 72 Xinmin Street, Changchun, 130021 Jilin China

**Keywords:** Umbilical cord stem cells, Osteoarthritis, Chondrogenesis, Regeneration engineering, Tissue engineering

## Abstract

Osteoarthritis is a chronic degenerative joint disease with an incidence of 81% among people aged over 65 years in China. Osteoarthritis significantly decreases the quality of life of patients, causing physical and psychological damage and posing a serious economic burden. Clinical treatments for osteoarthritis include drug and surgical treatments. Drug treatment can successfully alleviate pain but not satisfactorily reverse joint damage, while surgical intervention is typically used to treat end-stage disease. Stem cells are multi-potential progenitor cells with self-renewal and multi-lineage differentiation abilities, and can differentiate into many kinds of cells, including chondrocytes. Umbilical cord stem cells, also known as Wharton’s jelly mesenchymal stem cells (WJ-MSCs), have become the first choice for cartilage regeneration engineering owing to their availability and convenience of collection. This article reviews the biological characterization of WJ-MSCs in recent years, their advantages compared with other stem cells, and their application in the treatment of osteoarthritis in animal experiments and clinical trials.

## Introduction

Osteoarthritis (OA) is a common chronic joint disease characterized by degeneration, destruction, and osteoporosis of joint tissue. The occurrence of OA is related to aging, obesity, inflammation, trauma, overuse of joints, metabolic disorders, and heredity. OA is also termed degenerative arthritis or senile arthritis. At present, clinical treatment for osteoarthritis comprises drug and surgical intervention. Surgical intervention is typically used to treat end-stage osteoarthritis [1]. Drug therapy only alleviates pain during early osteoarthritis and does not reverse or treat the damage to articular cartilage. Stem cells are multi-potent progenitor cells capable of self-renewal and multi-lineage differentiation. Under conducive conditions, they can differentiate into various functional cell types, including nerve cells, osteocytes, and chondrocytes. Mesenchymal stem cells are found in synovium and solid joints, and can be isolated from synovium, meniscus, ligament, fat pad, and synovial articular cartilage. Thus, mesenchymal stem cells play an important role in the maintenance and function of these tissues [2–4]. Stem cell transplantation could be used to treat early osteoarthritis and reverse the disease progression. WJ-MSCs have recently been discovered in the human umbilical cord and extensively studied; these cells are a promising prospect for clinical regeneration engineering and the development of novel early interventions for OA. The purpose of this article is to summarize recent progress in research on the application of WJ-MSCs in osteoarthritis, and to highlight some challenges that we believe need to be overcome for the successful application of WJ-MSCs in osteoarthritis treatment.

## Biological advantages of WJ-MSCs

### Source

The umbilical cord (UC) is covered with squamous epithelium, which is a continuation of the amniotic membrane. The umbilical cord is composed of two arteries, one vein, and mucoid connective tissue (Wharton’s jelly) between them. Wharton’s jelly is a mucoid connective tissue rich in proteoglycans and hyaluronic acid, which insulates and protects the umbilical cord [5]. Romanov et al. [6] extracted stem cells with multi-lineage differentiation potential from Wharton’s jelly, which were termed Wharton’s jelly mesenchymal stem cells (WJ-MSCs).

### Morphological characteristics

WJ-MSCs grow adherently and appear similar to short fibroblasts when cultured in vitro, with parallel, spiral, reticular, and shooting growth patterns. They have abundant cytoplasm and large nuclei, unclear cell boundaries. After adherent WJ-MSCs are detached by trypsin digestion and passaged for a certain duration, cell morphology changes into a long spindle shape to form fibroid cells of uniform size. Under low power microscopy, the cells appear to grow in a spiral or unipolar radial shape, similar to that of bone marrow mesenchymal stem cells (MSCs) [7, 8]. WJ-MSCs could maintain stable proliferation in the undifferentiated state and proliferate in vitro for more than 10 generations. Thus, WJ-MSCs have the biological characteristics of easy culture and expansion. Zhang et al. [9] found that a primary culture of 1 × 10^6^ WJ-MSCs could yield 1 × 10^10^ WJ-MSCs after 4 weeks of expansion, which was sufficient for clinical use.

### Cell surface markers

Karahuseyinoglu et al. [10] showed that WJ-MSCs express CD10, CD73, CD49, CD166, CD90, and CD146 in addition to CD44, CD13, CD29, CD105; but not CD14 and CD34. WJ-MSCs also express surface markers related to transplant immune rejection, such as CD86, CD80, and CD40. The endothelial cell antigen CD31 is not expressed, which is consistent with the phenotype of bone marrow mesenchymal stem cells [8, 11]. Thus, WJ-MSCs likely have functions similar to those of bone marrow mesenchymal stem cells. The expression of SH2, CD106, and HLA-1 is lower in WJ-MSCs than in bone marrow mesenchymal stem cells [12], which may indicate that WJ-MSCs are a more primitive mesenchymal stem cell population and have a stronger ability to differentiate directionally.

### Immunogenicity

WJ-MSCs also play an important role in acquired immunity; they show low expression of MHC class I molecule, and do not express MHC class II molecule and costimulatory molecule necessary for T cell activation. Therefore, allogeneic WJ-MSCs do not induce T cell proliferation response [13]. Among stem cells from different sources such as bone marrow mesenchymal stem cells, adipose tissue-derived mesenchymal stem cells, and placental mesenchymal stem cells, the expression of MHC II and immune-related genes was the weakest in WJ-MSCs, which minimized the scavenging effect of NK cells. In co-culture experiments, WJ-MSCs had the most significant inhibitory effect on T lymphocytes. The immune system has good tolerance to WJ-MSCs [14], likely because of their low immunogenicity. Therefore, WJ-MSCs are potentially one of the best cell types for clinical tissue regeneration.

### Secretion characteristics

WJ-MSCs also have a paracrine effect; they can secrete a variety of biologically active factors to effect a specific biological function [15]. WJ-MSC exosomes include active secretions of cell-related miRNA, mRNA, and protein secreted by WJ-MSCs. They could function as important signal-transmitting substances between cells and participate in WJ-MSC-mediated damage repair and immune regulation. For example, WJ-MSC exosomes could promote lung adenocarcinoma growth by transferring miR-410 [16]. WJ-MSC exosomes could inhibit the expression and function of Th22 cells. Th22 are novel CD4+ helper T cells that secrete interleukin 22 and tumor necrosis factor alpha, and have a pro-inflammatory effect in many diseases such as tumors and rheumatoid arthritis [17]. Thus, WJ-MSCs exosomes play an important role in inhibiting inflammation.

## Advantages in the application of WJ-MSCs

### Availability

The collection of most stem cells (such as bone marrow stem cells) from donors requires invasive procedures, and large-scale extraction is typically not possible. However, the acquisition of WJ-MSCs is easier and non-invasive. Extraction of WJ-MSCs does not involve ethical issues; at present, most hospitals treat umbilical cord as medical waste, and the collection procedure is non-invasive. Thus, WJ-MSCs are abundant in origin, easy to collect, and have no adverse effects on the donors [18, 19].

### Culture characteristics

WJ-MSCs have good freeze–thaw properties and can be frozen in liquid nitrogen Dewar bottles long-term, and thawed when needed. This feature is convenient for basic experimental work on WJ-MSCs, and provides a good theoretical basis for the establishment of a “clinical resource bank” in the future [20].

### Low immunogenicity

WJ-MSCs also express the chemokine receptors CXCR3, CXCR4, and CXCR6, which play a key role in targeted migration of stem cells to tissue damage sites [21]. In recent years, the immunogenicity and immunosuppressive functions of WJ-MSCs through cell contact and paracrine mechanisms cells have been confirmed by in vitro studies of xenogeneic and allogeneic models. Pretreatment with pro-inflammatory cytokines could even improve the immune regulation of WJ-MSCs [22]. In addition, WJ-MSCs did not induce the proliferation of xenogeneic and allogeneic immune cells. The expression of the immunosuppressive human leukocyte antigens HLA-G6, IL-6, and VEGF and the absence of the costimulatory molecules CD40, CD80, and CD86 further support the immunosuppressive properties of umbilical cord stem cells [23].

### Differentiation potential

WJ-MSCs are superior to stem cells from other sources such as bone marrow in terms of osteogenic and chondrogenic differentiation ability [24]. Reppel et al. cultured WJ-MSCs on three-dimensional scaffolds without adding growth factors, and evaluated their chondrogenic differentiation ability at the transcriptional and protein levels. After 28 days of scaffold culture, the expression of cartilage-specific markers at the transcriptional level was significantly up-regulated. Further, WJ-MSCs exhibit more type II collagen synthesis than do bone marrow stem cells [14]. Because of the relative difficulty in obtaining bone marrow stem cells, WJ-MSCs have better application prospects.

## Current progress in the use of WJ-MSCs for the treatment of osteoarthritis worldwide

Recently, there have been several studies worldwide aiming to reverse the progression of osteoarthritis using stem cell-based regenerative strategies.

### In vitro basic experiments

Aleksander-Konert et al. [25] used hydrogel scaffolds to encapsulate WJ-MSCs and added appropriate cell culture medium after gelation. Chondrogenesis in the hydrogels was determined by Alcian Blue and Safranin O staining, and gene expression in the extracellular matrix (collagen type I, type II, type III and aggregated proteoglycan) was determined by real-time PCR. Alcian Blue and Safranin O staining showed that the WJ-MSCs encapsulated in hydrogel produced a large amount of extracellular matrix, which contained abundant proteoglycan (the main component of hyaline cartilage). The expression of collagen II and aggregating proteoglycan (the main marker for hyaline cartilage) increased in cultures containing chondrogenic media, which indicated that WJ-MSCs had a strong ability to differentiate into chondrocytes under these conditions. These results provided a good theoretical basis for future clinical applications of WJ-MSCs in the treatment of osteoarthritis.

In osteoarthritis (OA) pathogenesis, the RELA gene is involved in cartilage degradation via MMP-13. As a member of the NF-ĸβ gene family, RELA modulates inflammatory responses and activates pro-inflammatory cytokines. Sofia et al. [26] performed a study on synovial tissue from patients with OA undergoing total knee replacement (TKR) surgery as follows: The study comprised six groups treated with 4 replications. Group I and II (control groups) comprised synoviocytes from OA incubated for 24 and 48 h, respectively. Group III and IV comprised WJ-MSCs incubated for 24 and 48 h, respectively. Group V and VI were Synoviocyte-WJ-MSCs co-culture incubated for 24 and 48 h, respectively. MMP-13 and RELA gene expression in each group was detected by qPCR. The results showed that WJ-MSCs reduced MMP-13 gene expression after co-culture for 24 and 48 h in OA synoviocytes. Thus, WJ-MSCs could play an important role in delaying the progress of osteoarthritis.

### Animal studies

Wu et al. induced cartilage wear in the knees of age-, weight-, and sex-matched miniature pigs. Then, 1.5 mL of a WJ-MSCs (5 × 10^6^ cells) and hyaluronic acid (HA) hydrogel composite was then transplanted into the chondral-injured area in the right knee of each pig. The left knee was used as a control. After 12 weeks of transplantation, the degree of cartilage repair was determined by macroscopic and microscopic observation. The cartilage wear in the treatment group was significantly reversed compared to that in the control group. The histological score of the knee joint after treatment by the International Society of Cartilage Repair was higher than that of the control group [27].

Zhang et al. [28] explored the feasibility of using WJ-MSCs combined with acellular chondrocyte extracellular matrix (ECM) oriented scaffolds to construct tissue-engineered cartilage for repairing full-thickness cartilage defects in the weight-bearing area of the goat knee joint. The engineered cartilage complex with WJ-MSCs (containing 1 × 10^7^ cell suspension) was placed in articular cartilage defect of goats in the treated model group. The difference between the treatment and control groups was determined by observing the level of inflammatory reaction, morphological score, histological score, and quantity of glycosaminoglycan. H&E staining of knee joint fluid showed no significant difference between the treatment and control groups. Morphologically, 3 months after treatment, the size of the cartilage defect was significantly smaller in the experimental group than in the control group. New cartilage-like tissue covered the subchondral bone. The defect was well integrated with the edge of normal cartilage. After 6 months, the cartilage defect was completely covered by the new cartilage tissue. Histologically, a variety of staining methods were used to score the results. The treatment group showed better therapeutic results than the control group. Quantitative analysis of glycosaminoglycan showed significantly higher levels in the experimental group than in the control group. Thus, many studies have shown that the implantation of WJ-MSCs in animals does have a certain therapeutic effect on osteoarthritis. Corresponding clinical trials have also been carried out.

### Clinical trials

Wang et al. [29] conducted clinical trials on intra-articular injection of stem cells in the treatment of arthritis. Thirty-six patients with similar and no statistical difference were selected and randomly divided into two groups. Intra-articular injection of 2.5–3.0 mL WJ-MSC suspension containing (2–3) × 10^7^ cells was performed once a month, 2 times as a course of treatment in the cell treatment group; sodium hyaluronate was administered by intra-articular injection once a week, 5 times in the control group. Follow-up showed that although the incidence of pain and swelling in the treatment group was higher than that in the control group 1 month after injection, the scores were significantly better in the treatment group than in the control group. Thus, intra-articular injection of WJ-MSCs improved joint function. Intra-articular injection of WJ-MSCs can improve osteoarthritis in part; however, there is still limited information on the efficacy of intra-articular injection of WJ-MSCs in clinical outcomes and cartilage repair, as well as standardized evidence on some basic issues such as single injection and metering, injection frequency, and injection interval.

Sadlik et al. validated the regenerative effect of WJ-MSCs on cartilage by arthroscopic implantation of collagen scaffolds containing WJ-MSCs. They placed extracted WJ-MSCs on aluminum foil or aseptic latex dental plate templates. The scaffolds were placed in cartilage defects, and MRI images were monitored postoperatively and 1.5, 6, and 12 months after operation. A large amount of regenerated tissue was observed, and the surrounding cartilage and subchondral bone were fused. Thus, WJ-MSCs could be used as seed cells to induce cartilage regeneration in patients with osteoarthritis [30].

Ha et al. randomly selected 200 patients with moderate or mild osteoarthritis and divided them into four groups. They were injected with 5 mL pure platelet-rich plasma, 5 mL WJ-MSCs, 5 mL platelet-rich plasma combined with WJ-MSCs, or 5 mL hyaluronic acid sodium. To observe the short-term efficacy of platelet-rich plasma combined with WJ-MSCs intra-articular injection in the treatment of mild to moderate knee osteoarthritis, WJ-MSCs injection or simple sodium hyaluronate injection was used. Visual simulated pain score and American Knee Association score showed that sodium hyaluronate injection did not improve the knee joint after 3 months of treatment. The remaining groups showed remission, and the combined injection of platelet-rich plasma and WJ-MSCs showed significantly better results than did the other two groups. The expression of cytokines in MRI and synovial fluid was also improved. Therefore, platelet-rich plasma combined with intra-articular injection of WJ-MSCs has a significant therapeutic effect on the clinical symptoms of osteoarthritis [31].

Matas et al. evaluated the safety and efficacy of single or multiple intra-articular injections of WJ-MSCs in the treatment of osteoarthritis. Twenty-nine patients with knee OA were randomly sampled at baseline and 6 months (ha, *n* = 8), single dose (20 × 10^6^) WJ-MSCs (MSC-1, *n* = 9) at baseline or repeated WJ-MSCs doses at baseline and 6 months (20 × 10^6^ × 2; MSC-2, *n* = 9). During the 12-month follow-up, no adverse reactions such as death, neoplasia, or infection were observed. The pain and function of the patients injected with WJ-MSCs significantly improved, and the clinical score was significantly higher than that of the control group. There was no statistical difference in imaging results. Thus, multiple injections of WJ-MSCs showed good safety in the treatment of osteoarthritis, and had obvious clinical efficacy in the treatment of long-term pain caused by osteoarthritis [32].

## Conclusion

The study of osteoarthritis, particularly the use of stem cell therapy, in joint disease research has attracted considerable attention. Some precise studies have been carried out on the function and characteristics of WJ-MSCs and their application in cartilage regeneration to treat osteoarthritis. According to reports from basic research and clinical trials, it is safe and effective to use WJ-MSCs to treat osteoarthritis. However, there are several avenues that merit further study, such as (1) correlation between the degree and course of articular cartilage injury and the efficacy of stem cell therapy. (2) Induced signaling pathways and related mechanisms of directed differentiation of transplanted WJ-MSCs. (3) Design of in vitro experiments to artificially control and regulate the growth and differentiation of stem cells in vivo. (4) Design of clinical trials and standardization of the timing of injections and transplantation options. Therefore, in subsequent studies, we need to address the shortcomings of current research, lay a solid theoretical foundation for the clinical application of WJ-MSCs in the treatment of osteoarthritis, and possibly attempt the reversal of osteoarthritis.
